# Effectiveness and Feasibility of Nonpharmacological Interventions for People With Parkinson's Disease and Cognitive Impairment on Patient-Centred Outcomes: Systematic Review and Meta-Analysis

**DOI:** 10.1155/2024/3654652

**Published:** 2024-11-18

**Authors:** Jennifer S. Pigott, Megan Armstrong, Nujhat Tabassum, Nathan Davies, Anette Schrag

**Affiliations:** ^1^University College London, London, UK; ^2^Queen Mary University London, London, UK; ^3^Aberdeen Royal Infirmary, NHS Grampian, Aberdeen, UK

## Abstract

**Background:** Cognitive impairment is common in Parkinson's disease (PD) but has limited treatment options. Medication has shown some benefits but accompanied by risk of adverse events. We aimed to investigate effectiveness and feasibility of nonpharmacological interventions for people with PD and cognitive impairment on patient-centred outcomes.

**Methods:** Systematic searches of five databases (MEDLINE, Embase, CINAHL, PsycINFO and Web of Science) were performed for studies evaluating nonpharmacological interventions for people with PD and cognitive impairment, reporting health-related quality of life, function (activities of daily living) or wellbeing outcomes, published up to 15 May 2023. Two reviewers independently assessed full-text articles and one reviewer extracted data, with a second reviewer reliability checking all data extraction. Randomised controlled trials (RCTs) were synthesised through meta-analysis using a random-effects meta-analysis with restricted maximum likelihood method pooled estimate and observational studies through narrative synthesis.

**Results:** Eleven RCTs and three noncontrolled studies were included, studying a range of interventions: cognitive training, cognitive stimulation, cognitive rehabilitation, physical and cognitive exercise, goal management training, psychoeducation with mindfulness, broader rehabilitation programs and a psychological intervention. Feasibility was demonstrated. The majority showed effectiveness for their primary outcome. Meta-analysis showed no significant improvement in HrQoL (seven RCTs: pooled effect, standardised mean difference, −0.20 [−0.57−0.18]) or function (four RCTs: 0.08 [−0.36, 0.52]), and wellbeing measurement was infrequent and indirect. Quality of evidence was judged as very low, limiting the conclusions drawn.

**Conclusion:** Whilst nonpharmacological trials for cognitive impairment in PD have shown promise, we found no evidence of effectiveness on HrQoL, function or wellbeing. However, this is based on very low-quality evidence from a small number of diverse studies, not powered for these outcomes. Feasibility of a range of interventions has been demonstrated in both PD-mild cognitive impairment and PD-dementia. There is a need for more robust, adequately powered studies.

## 1. Introduction

Parkinson's disease (PD) is a multifaceted neurodegenerative condition, traditionally seen as a motor disorder, but with increasing recognition of nonmotor features such as depression, cognitive impairment, gastrointestinal symptoms and sleep disturbance [1]. Cognitive symptoms in PD exist across a spectrum of severity [2]: subjective cognitive decline to mild cognitive impairment (PD-MCI) to dementia (PDD) with diagnostic criteria set out by the Movement Disorders Society (MDS) [3, 4]. Research in PD-MCI and PDD is limited, particularly regarding management [5].

Severity of nonmotor symptoms and impairment of activities of daily living (ADLs) have been shown to be independently associated with worse health-related quality of life (HrQoL) in people with PD [6]. There is evidence that cognitive impairment contributes to poor HrQoL in PD [7–9]. The presence of dementia significantly decreases HrQoL, and overall functional impairment progresses with cognitive decline [8]. Education may be protective, potentially due to increased cognitive reserve, and the impact on HrQoL lessens with time, potentially mediated by adjustment and coping [10].

Most medical treatments (pharmacological or surgical) for PD target motor disturbances [11], leaving nonmotor aspects to continue to cause deterioration in daily life. This highlights the importance of complementary approaches to improve HrQoL. Regarding management, cholinesterase inhibitors and memantine improve cognition, global function, ADLs and neuropsychiatric symptoms, but with an increased rate of adverse events, compared to placebo, so are recommended for use in PDD [12], although the role of medication in PD-MCI is less clear. Cognitive impairment is seen as an unfavourable factor in selection for PD surgical treatments, such as deep brain stimulation for a variety of reasons, mostly related to increased risk [13]. However, nonmedical management options are not well established.

Cognitive interventions include cognitive training, cognitive stimulation and cognitive rehabilitation. Cognitive training is the guided practice of tasks targeting particular cognitive domains, aiming to improve those cognitive functions [14]. Cognitive stimulation is engagement in a range of activities and discussions (usually in a group) aimed at the general enhancement of cognitive and social functioning [15]. Cognitive rehabilitation is a personalised, goal-directed problem-solving approach, aimed at improving functioning in everyday life, utilising enhanced learning methods and compensatory strategies [16]. These interventions have been explored in dementia more broadly [14–16], with some positive findings, but poor quality evidence frequently noted. There is also a dearth of evidence regarding the effect of these interventions in MCI [17–19]. There is a suggestion that physical activity and cognitive exercise may improve some cognitive functions for people with MCI, but more research is needed [20].

In PD, there is a paucity of studies evaluating nonmedical interventions for those with cognitive impairment [21]. A systematic review of nonpharmacological therapies for the enhancement of cognitive function in PD identified studies of cognitive training; exercise and physical therapies; combined cognitive and physical interventions; and brain stimulation techniques [22]. Improvements were seen for a range of cognitive domains in most studies, some compared to controls and some of pre–post design, but most participants were not cognitively impaired, and none had dementia. Quality of studies was generally low, and publication bias was suspected. A more recent review of cognitive training for people with PD and dementia or MCI found no good evidence that this intervention is helpful, but studies were considered small and flawed [23]. Research has focussed on improvements in cognitive performance on neuropsychological tests, but the ultimate aim of these interventions is a meaningful benefit for patients in their daily lives. Whether nonpharmacological interventions can improve patient-centred outcomes, including HrQoL, wellbeing and day-to-day function in this population has not been established.

The primary aim of this review was to establish the effectiveness of nonpharmacological interventions in people with PD and cognitive impairment from a patient-centred perspective: focussing on HrQoL, wellbeing and function (ADLs) outcomes. The secondary aim was to investigate feasibility of interventional studies in this population.

## 2. Materials and Methods

### 2.1. Source of Data and Search Strategy

The Preferred Reporting Items for Systematic Reviews and Meta-analyses (PRISMA) guidelines [24] were followed. The review protocol was registered on PROSPERO: CRD42023213998.

Online database searches were conducted in MEDLINE, Embase, CINAHL, PsycINFO and Web of Science from inception to 26 April 2020 and an updated search was conducted 15 May 2023, with forwards and backwards citation searching. Searches were not restricted by language or date of publication.

The search strategy involved a combination Parkinson's terms; AND Cognitive impairment terms; AND support intervention terms; AND quality of life, wellbeing and function outcome terms. The full search strategy is available in the supplementary materials.

### 2.2. Inclusion and Exclusion Criteria

Peer-reviewed original quantitative research studies were eligible. Expert opinions, letter to the editor, case reports and case series, reviews, editorials, conference abstracts without full report and exclusively qualitative studies were excluded.  Table 1 shows the full inclusion and exclusion criteria. We included studies where caregivers participated, but addressed patient outcomes. Articles were excluded if the full text was not available in the English language.

### 2.3. Study Selection

Title and abstracts were screened for inclusion by one reviewer (NT or JP), with a 10% sample screened independently by the other for reliability. Full texts were reviewed independently by both reviewers (NT and JP). Any discrepancies were discussed and resolved with a third reviewer (MA or AS).

### 2.4. Data Extraction

JP extracted data and conducted the quality assessment; all checked for accuracy by other authors (MA and NT). Data were extracted into a standardised form, including lead author, publication date, country; population; study design; intervention type; sample size, participant age, PD stage and cognition; primary outcomes; and results for measures of interest for this review.

### 2.5. Data Synthesis

Meta-analysis was conducted for the randomised controlled trials (RCTs) that had sufficient homogeneity (similar aims, interventions and outcomes). Data were synthesised for the immediate postintervention outcome and if there was 3–6 months postintervention outcome. We estimated the standardised mean difference (Hedges' g) and standard error from each study, then used random-effects meta-analysis with restricted maximum likelihood (REML) to estimate the pooled estimate. Authors of the primary studies were contacted for any missing data. Heterogeneity was quantified using the *I*^2^ statistic and was investigated through post hoc subgroup analyses. Statistical analyses were conducted using Stata 17.0 [25]. A narrative synthesis approach was taken for those not included in the meta-analysis.

### 2.6. Quality

The GRADE method [26] was applied for reporting quality of evidence for each outcome. Potential bias within RCTs was assessed using the Cochrane Risk of Bias 2 (RoB2) tool [27]. Nonrandomised studies of interventions (NRSIs) were assessed using the ROBIN-I tool [28].

## 3. Results

### 3.1. Study Selection

**Figure 1:**
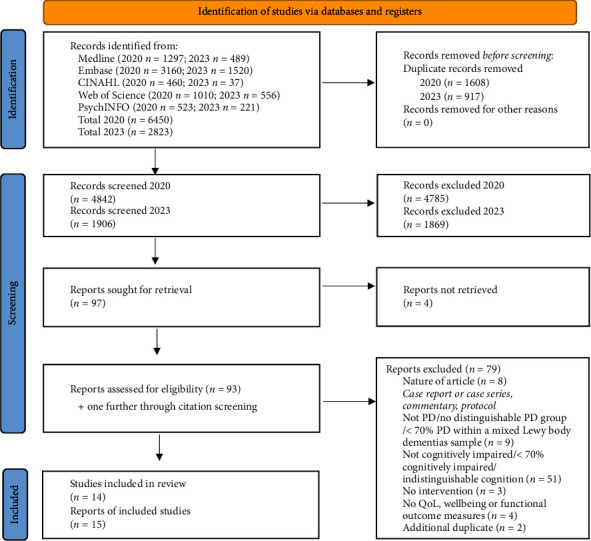
Prisma flowchart for search results. Abbreviations: PD, Parkinson's disease; QoL, quality of life.

As shown in Figure 1, the online database search yielded 6450 articles in 2020 and 2823 in 2023. A total of 14 studies were included following screening.

### 3.2. Study Characteristics

Eleven RCTs (two using a crossover design) and three noncontrolled studies were included. Five hundred and eighty participants with PD and cognitive impairment (or with > 70% of the study sample having cognitive impairment), plus 25 participants with DLB, are included across the RCTs, with a further 78 participants with PD and cognitive impairment in the non-RCTs. Most studies were small or had small subgroups with cognitive impairment. Cognitive function of participants varied between studies due to the different inclusion criteria (Table 2) and reflected in the objective cognitive assessments where reported (see Supporting Information (available here)). The MDS diagnostic criteria were frequently applied, with others using cognitive symptoms or assessments to distinguish those with cognitive impairment.

The selection of outcome measures and control arms varied greatly. No studies used our outcomes of interest as their primary outcome and were therefore not powered for these outcomes. The results of the primary outcomes and outcomes of interest for the RCTs are presented in Table 2.

### 3.3. Interventions

Various interventions were evaluated: cognitive training (four studies; one of which included it with and without transcranial direct current stimulation (tDCS), one combined it with physical rehabilitation); cognitive stimulation (two studies); cognitive rehabilitation (three studies; one combined it with strategy training; one was multimodal combining cognitive training, psychomotor training and transfer training); combined cognitive and physical exercise (one study); goal management training and psychoeducation with mindfulness (one study with two intervention arms); broader rehabilitation programs (two studies); and a psychological intervention (one study). The interventions are described in Table 3, detailing the components using the TIDieR checklist [45].

Where specified, sessions ranged from 30 min to 2 h in duration (mean 63 min) at a frequency between once a week to 10 times a week. The mean total number of sessions was 16, but ranged from 5 to 39, with some also involving ‘homework' tasks in addition. Total ‘dose' ranged from 375 to 2700 min (where ranges were given the midpoint has been used), with a mean of 955 min. Intervention duration ranged from 4 to 14 weeks, with one further adding a continuation at home phase [39]. Delivery of interventions also varied: Five were computer-based (including one virtual reality device), four were group-based, four were in-person individual therapist-delivered, and one was delivered by care partners at home. Many of the interventions had been tailored or adapted to PD, though others did not specify this.

### 3.4. Comparators

Different comparators were included in the RCTs: Nine used active controls (physical activity or rehabilitation; one including psychoeducation; relaxation therapy; cognitive training) and four used treatments as usual (two multiarm trials used both). Two noncontrolled studies used a pre–post design and one trial compared participant groups (PD with cognitive impairment and PD with normal cognition), and therefore, only pre–post findings for the cognitively impaired sample are included as relevant to this review.

### 3.5. Quality Assessment

According to the RoB-2 tool, two RCTs were deemed to have low risk, three had some concerns, and six were assessed as high risk of bias. The source of bias was frequently attributable to blinding of participants, personnel and outcome assessors, especially since these outcome measures are predominantly participant-reported. Concerns of potential bias arising from randomisation procedures or potential carryover in crossover trials were raised for five RCTs. Heterogeneity was moderate to substantial, and precision of effect estimates was generally low. Publication bias was suspected based on asymmetry of the funnel plots, though unfortunately, full data were not available for all studies. See Supporting Information for details of the risk of bias assessment and GRADE evaluation of evidence.

### 3.6. Effectiveness of Interventions

The evidence is summarised using the GRADE approach in Table 4 and individual RCT findings in Table 2.

Although none of the studies used our outcomes of interest as their primary outcome, the majority (10 out of 11) showed effectiveness or feasibility according to their aim. Three of the four RCTs investigating cognitive training interventions evaluated cognition as the primary outcome, all being effective [29, 32, 33], and the other did not specify a primary outcome but also showed improvements in cognition [31]. The two RCTs investigating cognitive stimulation, both including participants with PDD, were pilot studies primarily evaluating feasibility and were found to be feasible, though neither showed significant effects on cognition [34–36]. Cognitive rehabilitation and multimodal interventions combining physical and cognitive components used more varied primary outcome measures: goal attainment and satisfaction [37], cognition [39], balance [40] and social participation [38]. The first three of these were effective on their selected outcome compared to controls; the last of these was not significant, though was compared to an active control (cognitive training). The multimodal cognitive rehabilitation intervention showed the superiority of the three-component intervention (cognitive training, transfer training and psychomotor training) over both control groups, and superiority of the cognitive training plus transfer training control group compared to a cognitive training only control group, as well as pre–post improvements for the cognitive training only control group, suggesting a benefit from all three components [39]. One RCT compared two active interventions, goal management training and psychoeducation with mindfulness, showing significant pre–post improvement in executive function for both but no significant difference between them.

#### 3.6.1. HrQoL

All 11 RCTs measured HrQoL, with four reporting a significant improvement. One RCT found a significant effect of cognitive training on HrQoL compared to controls (6-arm study) [33]. One RCT found a significant effect of cognitive rehabilitation on QoL and HrQoL compared to active and inactive controls [37]. One RCT found significant effect of a multimodal (cognitive, transfer and psychomotor training) intervention on six of eight domains of HrQoL (mobility, ADLs, emotional perception, stigma, social support and cognition), compared to the active control arms [39]. Another RCT showed significantly improved HrQoL following psychoeducation with mindfulness compared to goal management training (no control arm) [41]. The other RCTs did not report significant effects of cognitive training [29–32], cognitive rehabilitation [38], cognitive stimulation [34, 35] and combined physical and cognitive exercise [40] on HrQoL compared to controls. The other two showed significant pre–post improvements: one for improved coping following computer-assisted virtual reality rehabilitation [43] and the second showed improved disability following a self-directed internet-based psychological course for chronic neurological conditions (PD subgroup) [44].

**Figure 2:**
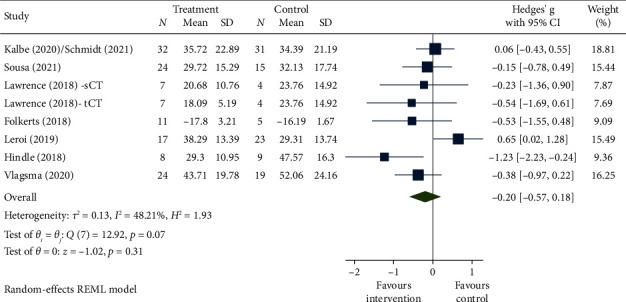
Forest plot for HrQoL immediately postintervention.

Meta-analyses were conducted on seven RCTs to evaluate the effect of the intervention on HrQoL immediately postintervention. As shown in the forest plot (Figure 2), there was no evidence of effectiveness postintervention with regard to HrQoL, with pooled effects and Hedges' *g* [95% confidence interval] of −0.20 [−0.57–0.18] (negative indicates improvement). Follow-up times varied, but five RCTs were included in the meta-analysis of medium-term follow-up (3–6 months postintervention), which similarly did not show a significant pooled effect: −0.25 [−0.73, 0.23] (forest plot provided in Supporting Information).

#### 3.6.2. Function

Seven RCTs measured function with one, evaluating cognitive training, reporting significant improvement, compared to controls (6-arm study) [33]. For the noncontrolled studies, two measured functions: One showed improved disability following a self-directed internet-based psychological course for chronic neurological conditions (PD subgroup) [44].

**Figure 3:**
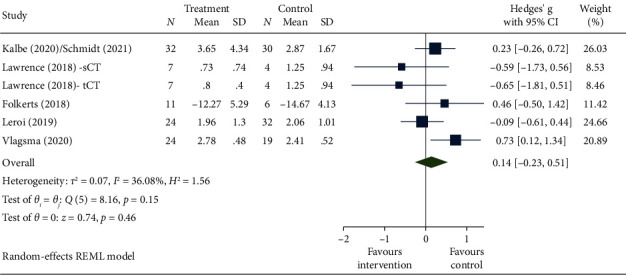
Forest plot for function immediately postintervention.

Meta-analyses were conducted on five RCTs to evaluate the effect of the intervention on function immediately postintervention (Figure 3), showing no significant pooled effect: Hedges' *g* 0.14 [−0.23, 0.51] (positive indicates worsening). Meta-analyses of four RCTs evaluating a 3- to 6-month follow-up were similarly nonsignificant: Hedges' *g* −0.16 [−0.51, 0.18] (forest plot provided in supporting information).

#### 3.6.3. Wellbeing

No RCTs measured wellbeing, though some measured related constructs (Table 2). For the noncontrolled studies, one showed significant pre–post improvements for improved coping following computer-assisted virtual reality rehabilitation [43].

### 3.7. Heterogeneity of the RCTs

There may be moderate heterogeneity: *I*^2^ = 48% and 36% for HrQoL and function, respectively, for immediately postintervention measurements, and 52% and 0% for follow-up. To investigate heterogeneity, subgroup analyses were performed (forest plots provided in the supporting information). This showed differences by intervention type (cognitive training, cognitive rehabilitation and cognitive stimulation) and comparator type (active vs. inactive controls), but group differences were not statistically significant. Results were more consistent (showing no effect) for HrQoL outcomes for studies including participants with MCI, but those including participants with dementia had mixed results (though note these also include different interventions).

### 3.8. Feasibility of Intervention Studies

Recruitment appeared nonproblematic for PD-MCI, but somewhat more challenging for PDD. Recruitment rates (calculated as participants assessed at baseline/potential participants invited—participants not meeting inclusion criteria) ranged from 39% to 75% for those including PDD [34–37], with one not recruiting the planned sample size [37], but 67%–100% for PD-MCI (reported in 5 studies [29, 32, 33, 38, 41]). Retention was generally good across the studies (> 70% for all), with the lowest being in a study inclusive of participants with dementia (26% attrition) [35]; retention in all others was > 80%. Where reported, concordance (see Table 3) and acceptability of interventions in this population were good. Insufficient concordance with the intervention such that the recommended dose was not achieved was reported for the intervention delivered at home by caregivers, to a mixed sample of PD-MCI, PDD and DLB [35].

## 4. Discussion

This systematic review identified 11 RCTs of nonpharmacological interventions for people with cognitive impairment in PD. The paucity of studies including participants with PDD (only three) is notable, especially given the relative wealth of evidence in dementia more broadly (nonspecified type or Alzheimer's disease) [14–16, 18].

Overall, whilst the majority showed effectiveness on the selected primary outcome, often a cognitive measure, there was no significant effect of cognitive interventions on HrQoL or function detected postintervention (seven and four RCTs, respectively), nor at 3- to 6-month follow-up (five and four RCTs, respectively). Wellbeing was only indirectly reported in two RCTs, preventing conclusions regarding the effects of nonpharmacological interventions on participant wellbeing.

The included studies were generally small, of low quality and highly diverse in terms of intervention type, dose and delivery as well as studies using varied samples, timepoints and comparators. Different targets were used, some focussing on improving cognitive function, others targeting the impact of cognitive impairment. Some interventions were multimodal, whereas others used cognitive training alone. Meta-analyses therefore need to be treated with caution. Since HrQoL, function and wellbeing outcomes were not the primary outcomes, none of the studies appear to have been powered to detect change in these measures. Larger trials are needed, powered to detect change in these patient-centred measures.

Our subgroup analyses, conducted to investigate heterogeneity rather than being a priori planned analyses, did not show statistically significant differences according to intervention type, control (active or inactive) or sample (PD-MCI and PDD combined, or PD-MCI alone). However, heterogeneity was lessened, suggesting that these factors play a role. With regard to intervention type, the cognitive training group in particular showed reduced heterogeneity when separated from other intervention types and findings were consistent with a systematic review and meta-analysis of cognitive training in PDD and PD-MCI, which found no good evidence of beneficial effects, reporting on cognitive and function outcome measures [23]. They similarly concluded that studies were small and flawed. Cognitive training is designed to target cognitive function specifically (in some cases domain-specific). Whilst cognitive impairment is associated with disability and reduced HrQoL in PD [8], the relationship between cognitive *improvement* and function and HrQoL is less clear. Other cognitive interventions, such as cognitive stimulation and cognitive rehabilitation, take a broader approach with more person-centred targets. These were evaluated by fewer studies with more heterogeneous results and have similarly not provided clear evidence of beneficial effects, but there appears a greater tendency towards benefit in the HrQoL outcomes. This is interesting considering findings from the dementia field more broadly (across dementia subtypes, but dominated by Alzheimer's disease), in which cognitive training has been seen to have beneficial effects on cognitive performance, but with insufficient evidence for the effect on disease severity or function [14]. However, there is evidence for cognitive stimulation for improving both cognitive performance and QoL. There is also evidence for the effectiveness of cognitive rehabilitation in terms of goal attainment, self-efficacy and some cognitive domains, but insufficient evidence regarding QoL and functional outcomes, though the evidence base is much smaller for cognitive rehabilitation [16].

Other interventions that could not be included in the meta-analysis included psychological interventions, goal management training (overlapping with cognitive rehabilitation) and combined physical and cognitive exercise. There was a suggestion of benefit for psychological interventions: psychoeducation with mindfulness being superior to goal management training [41] and pre–post improvement following a course integrating cognitive behavioural therapy with compensatory cognitive rehabilitation therapy [44]. In PD more broadly (not cognitive impairment), physical exercise has been shown to have beneficial effects not only on motor function but also on QoL [46]. A systematic review of group-based arts therapies, including dance, singing, music and theatre, found effectiveness on patient-centred outcomes including QoL and function, suggesting a more holistic approach may be beneficial [47]. Social aspects were incorporated in many of the included studies though less explicitly: through psychosocial or social cognition as targets, or through group delivery. It seems likely that interventions combining physical, cognitive, psychological and social components would be more likely to improve QoL, which is a multidimensional concept, though of course cost may become a barrier with greater intensity and complexity of intervention.

Studies varied in terms of timing of assessments and interventions varied in duration. Most interventions were relatively short (all 4–14 weeks; one added a continuation at home phase [39]) which may limit their effectiveness. Interventions rely on brain plasticity [48], and plastic changes are experience-dependent [49, 50] and relate to the activities being carried out and the state of brain health. These changes will therefore take time to be established (especially in the context of cognitive impairment in PD), so short-duration interventions are less likely to achieve or sustain the requirement changes. It is interesting to note that pilot studies of active theatre therapy in PD (not cognitive impairment specifically) had a long duration of intervention (1.5 years and 3 years) and were seen to be effective on patient-centred outcomes [51, 52].

Variation was seen in relation to the nature of the comparator used by the studies. Some RCTs lacked clear control arms, limiting comparisons between studies. However, as noted above, active control arms do offer the potential for blinding of participants, if they do not know which intervention is the one under primary investigation. We suggest that active dose-equivalent control arms that do *not* contain components suspected to mediate effect for the intervention of interest are most valuable in evaluating effect, and inactive controls allow greater potential for comparison and pooling of data between studies—for example, Hindle et al. used a relaxation therapy control arm in addition to a usual care control arm [37].

Despite the limited evidence of effectiveness, these studies are useful in illustrating feasibility of studying the effect of nonpharmacological interventions in patients with PD and cognitive impairment. Studies involving participants with PD-MCI showed high recruitment and retention as well as high concordance with the interventions. Studies appeared more challenging when including participants with PDD and DLB, with lower recruitment and retention rates; however, only one did not recruit the intended sample size [37], and only one reported not meeting the recommended ‘dose' [35]. These were however pilot studies. Of note, the latter was an intervention delivered by care partners in their own homes with telephone support, rather than being delivered by professionals, which may perhaps have contributed to the low concordance. Though it is only one study with many factors differing from the others, this raises the question of whether professional delivery may confer greater concordance, which has associated cost implications. Caregiver-delivered cognitive stimulation has been shown to be both deliverable and effective compared to usual care in dementia (not PDD-specific) [53], but we are not aware of any evidence comparing effectiveness between delivery methods.

### 4.1. Strengths and Limitations

The reproducible and robust methodology, following PRISMA guidelines, involving multiple databases and extensive search terms, and two independent reviewers are strengths of this review. The broad research question allows for expansive oversight of patient-important outcomes with a comprehensive overview of interventions, but the downside is the resulting heterogeneity of studies and interventions, limiting quantitative synthesis. Funnel plots for RCTs raise concern regarding publication bias, with possible overrepresentation of small positive studies. Overall, the quality of the evidence is poor, limiting interpretation of findings.

### 4.2. Implications

Further large high-quality RCTs are needed to evaluate the effectiveness of nonpharmacological interventions for people with PD and cognitive impairment, which appear to be feasible in this population. Areas for specific consideration in future trials should be the involvement of an active control arm to facilitate participant blinding. Concordance in intervention delivered via remote methods warrants further investigation. Regarding patient-centred outcomes, we recommend that cognitive stimulation, cognitive rehabilitation and multimodal interventions be further investigated. It would also be helpful to investigate interventions of longer duration and dose responses to elucidate the active components of complex interventions, as well as to inform the optimal dose for subsequent cost-effectiveness evaluation.

## 5. Conclusions

This review found no evidence that cognitive interventions (training, stimulation or rehabilitation) lead to improved HrQoL, function or wellbeing for people with PD and cognitive impairment. However, this conclusion is based on very low-quality evidence from a small number of highly diverse studies. Insufficient evidence was available for other nonpharmacological interventions. However, a range of interventions have been demonstrated to be feasible for both participants with PD-MCI and PDD. There is a need for more robust, adequately powered studies before conclusions can be drawn about the effectiveness of nonpharmacological interventions for this population.

## Figures and Tables

**Table 1:** Inclusion and exclusion criteria.

	Inclusion	Exclusion
Population	Adults with idiopathic PD and cognitive impairment (of any severity), with or without their carers	PD data not distinguishable from other conditions. Atypical or symptomatic parkinsonism.
	Where studies included broader samples of Lewy body dementias (including DLB as well as PDD) or a spectrum of cognitive function (inclusive of PD with normal cognition), we required >70% of the sample to be those with PD and cognitive impairment	

Intervention	Support or therapeutic interventions	Pharmacological interventions; invasive medical or surgical interventions (e.g., deep brain stimulation, transcranial stimulation)
		No intervention described

Comparator	Any	Nil

Outcome	Measures of QoL/HrQoL, function/ADLs and/or wellbeing	Absence of QoL/HrQoL, function/ADLs and/or wellbeing outcome measures

**Table 2:** Summary of RCTs.

Author (Year) country	Intervention and comparator	Sample		Assessment timepoints	Results				Summary of key findings
					*Mean (sd) for postintervention (< 2 weeks) timepoint unless otherwise stated*				
		Size, age, sex, stage of PD	Cognitive criteria		HrQoL outcomes	Wellbeing outcomes	Functional outcomes	Primary outcome	
Kalbe et al. [29] & Schmidt et al. (2021) [30] Germany	Cognitive training (I) versus physical activity training with psychoeducation (C); Duration: 6 weeks	*N* = 64 I: 67.7 years (7.2), 72.7% M C: 67.5 years (8.3), 51.6% M H & Y: 98% Stage I–III	PD-MCI by MDS Level II criteria and subjective cognitive impairment and/or MoCA < 2 6. Excluded PDD	Baseline; postintervention; 6 months; 12 months	*PDQ39* b I: 35.72 (22.89); C: 34.39 (21.19) Time–group interaction nonsignificant	Not measured	*Bayer activities of daily living scale* b I: 3.65 (4.34); C: 2.87 (1.67) Time–group interaction nonsignificant	Executive and memory functions—effectiveness seen for executive postintervention and memory at 6 but not 12 months	No significant change in HrQoL or ADLs. Lower baseline predicted training gains except for QoL
Sousa et al. [31] Brazil	Cognitive training (I) versus general rehabilitation programme (C) Duration: 4 weeks.	*N* = 39 I: 60 years (7.5), 83% M, 13% H & Y Stage III, Rest I–II C: 58.5 years ACT (9.8), 87% M, 7% H & Y Stage III, Rest I–II	PD-MCI according to MDS Level II diagnostic criteria	Baseline; postintervention	*PDQ39* b I: 29.72 (15.29); C: 32.13 (17.74)^c^ Change in score: I: t = 2.275, **p**=0.0229 C: t = 1.889, **p**=0.0588	Not measured	Not measured	Not specified	Pre–post improvement in HrQoL for intervention group. Significance of between-group changes is not reported.
Bernini et al. [32] Italy	Cognitive training + physical rehabilitation (I) versus standard physical rehabilitation (C) Duration: 4 weeks.	*N* = 41 I: 71.2 years (7.0), 30% M, H & Y 2.8 (0.96) C: 69.3 years (7.7), 61% M H & Y 2.9 (0.47)	PD-MCI single or multiple domains but must include executive domain on level II criteria	Baseline; postintervention; 6 months	*PDQ-8* b at 6 months I: 39.7 (21.4); C: 39.2 (21.6)	Not measured	Not measured	Cognition–effective.	No significant effect for HrQoL at 6 months.
Lawrence et al. [33] Australia	Standard cognitive training (sCT) versus tailored cognitive training (tCT) versus sCT + tDCS versus tCT + tDCS versus tDCS alone versus control (no intervention) (C) Duration: 4 weeks	Total *n* = 42; *n* = 21 included in this review (CT and control) sCT: 68.1 year (8.7), 57% M tCT: 65.6 years (5.2), 43% M C: 72.3 years (6.2), 43% M H & Y not reported	PD-MCI (MDS Level II criteria); cognitive deficits that did not interfere with functional independence	Baseline; postintervention; 12 weeks	PDQ-39^b^ sCT: 20.68 (10.76); tCT: 18.09 (5.19); C: 23.76 (14.92) Interaction⁣^∗^ (*F* = 2.96, *p*=0.003): improvement in sCT (*F* = 7.21, *p*=0.001) and tCT (*F* = 12.48, *p* < 0.001) only	Not measured	*UPDRS-II* b sCT: 0.73 (0.74); tCT: T1 0.80 (0.40); C: 1.25 (0.94). Interaction⁣^∗^ (*F* = 1.96, *p*=0.04): improvement in sCT only (*F* = 11.29, *p* < 0.001) and sCT + tDCS (*F* = 3.40, *p*=0.04)	(Implied) Cognition and ‘practical outcomes'—cognitive training effective for cognition	*Significant improvement for HrQoL* with sCT and tCT compared to other arms. *Significant improvement in* ADLs with sCT (with and without tDCS) compared to other arms
Folkerts et al. [34] The Netherlands	Cognitive stimulation (I) versus usual care (C) Duration: 8 weeks	*N* = 12 I: 76.7 years (5.6), 83% M, H & Y Stage II–III 33%, stage > III 67% C: 76.5 (8.9), 83% M H & Y Stage II–III 50%, > III 50%	Residents of PDD-specific long-term care unit; MMSE 10–25	Baseline; postintervention; 6 weeks. Crossover design (so no control at 6 weeks follow-up)	*EQ-5D-5L Index* a c I: 0.59 (0.31); C: 0.67 (0.30) *EQ-5D-5L VAS*^a^^c^ I: 63.50 (11.50); C: 50.83 (26.35) *QUALIDEM total*^a^^c^ I: 17.80 (3.21), C: 16.19 (1.67)	Not measured	*Median* *Barthel Index* a c I: 12.27 (5.29); C:14.67 (4.13)	Feasibility–Feasible. ‘Main outcomes': global cognition, neuropsychiatric symptoms, depression, ADLs, QoL, nonsignificant.	No significant group difference for QoL or ADLs.

Leroi et al. [35] UK	Cognitive stimulation (I) versus treatment as usual (C) Duration: 12 weeks.	N = 76.15 PD-MCI, 40 PDD, 21 DLB Median (IQR) I: 74.5 years (68–77), 79% M C: 75 years (72–81), 79% M H & Y not reported	MDS criteria: PD-MCI: Level 1 criteria; PDD: Probable or possible; DLB: Probable or possible	Baseline; postintervention	*Change from baseline, mean (sd)*			Feasibility measures– feasible, acceptable and well-tolerated [36]	No significant changes for HrQoL, wellbeing or function
					*PDQ-39* b I: 38.29 (13.39); C: 29.31 (13.74) Adj mean diffd (I:C) 0.91, *p*=0.382 *EuroQoL Index*^a^ I: 0.57 (0.32); 0.57 (0.32) *Adj mean diff*^d^ (I:C) = 0.05, *p*=0.241*EuroQoLVAS*^a^ I: 62.30 (17.94); C: 62.35 (22.22) *Adj mean diff*^d^ (I:C) = 1.75, *p*=0.370	*Relatives stress scale (RSS)* I: 32.48 (9.69); C: 35.20 (7.02) Adj mean diffd (I:C) −1.25, *p*=0.215 *Brief resilience scale (BRS)* I: 20.33 (4.89); C: 20.97 (5.18) *Adj mean diff*^d^ (I:C) = −1.17, *p*=0.174	*Pill questionnaire* b I: 1.96 (1.30); C: 2.06 (1.01) Adj mean diff^Ϯ^ (I:C) −0.05, **p**=0.435		
Hindle et al. [37] UK	Cognitive rehabilitation (CR) versus relaxation therapy (RT) versus treatment as usual (TAU) Duration: 8 weeks.	*N* = 29 I: 75.8 years (6.6), 80% M RT: 74.9 years (6.9), 70% M TAU: 78.6 years (5.8), 89% M 90% H & Y Stage I–III; 10% Stage IV	PDD or DLB diagnosed according to Movement Disorders Society consensus criteria and ACE-III ≤ 82	Baseline; postintervention (T1); 6 months (T2)	*WHOQOL-BREF* a -physical CR 12.5 (3.12); RT 12.33 (18.87); TAU 10.26 (2.69). *p* > 0.05 (T1 and 2) - *psych* CR 13.25 (8.82); RT 12.44 (2.13); TAU 10.44 (2.79), *p* > 0.05 (T1 and 2) - *environ* CR 16.13 (2.23); RT 15.33 (1.22); TAU 14.99 (2.38), *p* > 0.05 (T1 and 2) - *social*⁣^∗^ CR 15.85 (2.31); RT 14.78 (3.27); TAU 14.06 (4.30) ES d (95% CI) at T1: CR versus TAU 1.11 (0.09–2.14) *p*=0.039, CR versus RT 1.13 (0.1–2.16) *p*=0.037 (T2 *p* > 0.05) *PDQ-8*^b^ CR 29.3 (10.95) RT 31.94 (10.34) TAU 47.57 (16.3) T1: *p* > 0.05 [T2⁣^∗^: *F*(1,18) = 5.2, *p*=0.02)] *EQ5D3L*^a^*(T2) -index*⁣^∗^ CR 0.59 (0.31); RT 0.56 (0.31); TAU 0.13 (0.26) *F*(1,18) = 5.23, *p*=0.02, ES d (95% CI) CR versus TAU 1.74 (0.59–2.9) *p*=0.007 -*VAS* CR 67.86 (17.53); RT 57.22 (18.56); TAU 46.11 (17.82)	*Generalised self-efficacy scale (GSES)* a CR 31.5 (4.24); RT 28.22 (5.56); TAU 28.86 (2.5) ES d (95% CI): CR versus RT 1.07 (0.06–2.09) *p*=0.041^∗^	Modified-functional activities questionnaire (FAQ)^b^ (T2) CR: 13.57 (7.87); RT: 13.44 (8.29); TAU: 17 (8.59). Nonsignificant	Goal Attainment and satisfaction with goal attainment–effective	Significant improvement for QoL and self-efficacy with intervention compared to controls. For postintervention: WHOQOL-BREF social and self-efficacy significant. For 6-month follow-up: HrQoL (PDQ-8) and subjective health status (EQ-5D-3L) significant; ADLs not significant.

Vlagsma et al. [38] Netherlands	Cognitive rehabilitation with strategy training ‘ReSET' (I) versus cognitive training *‘CogniPlus'* (C) Duration 7–14 weeks.	*N* = 43 I: 60.2 years (10.4), 58% M, H & Y 2.4 (0.6) C: 62.6 (8.8), 68% M, H & Y 2.2 (0.4)	Problems in executive function in everyday life: semistructured interview and/or ≥ 18 on DEX + impairment on EF tests	Baseline; 2 weeks postintervention; 3–5 months	*PDQ-39* b I: 43.71(19.78) C: 52.06(24.16) Time–group interaction: *f* = 0.57 *p*=0.454, *n*_*p*_^2^ = 0.01	Not measured	*Brock adaptive functioning questionnaire (BAFQ)* b [Self-score] I: 2.78 (0.48); C: 2.41 (0.52) Time–group interaction: *f* = 0.00, *p*=0.969, *n*_*p*_^2^ = 0.00	Role Resumption list (participation in different societal domains)–no significant differences.	No significant difference in function or HrQoL
Reuter et al. [39] Germany	Inpatient multimodal cognitive rehabilitation: Cognitive training + transfer training + psychomotor and endurance training (I) versus cognitive training (C1) versus cognitive training + transfer training (C2) Duration: Active rehab Phase 4 weeks; home continuation Phase 6 months	*N* = 240 All: 64 years (4) I: 53% M, 89% H & Y II–III, others IV C1: 51% M, 87% H & Y II–III, others IV C2: 52% M, 87% H & Y II–III, others IV	PD-MCI: Cognitive decline symptoms, preferably corroborated, end cognitive abnormalities which cannot be simply attributed to age but with minimal effect on daily function and no dementia	Baseline; post-inpatient intervention; at 6 months (after home training)	*6 months: PDQ-39* b Numerical outcome data not provided. % Of participants showing improvement: 13.8% (C1), 38% (C2), 52% (I). Group difference significant (in favour of intervention) for mobility and ADL domains (*p* < 0.001), emotional perception, stigma and cognition (*p* < 0.01) and social support (*p* < 0.05)	Not measured	Participants and caregivers reported improvement in ADLs in an evaluation of the intervention but no specific ADL measure reported.	ADAS-COG. All groups improved significantly, with a significant group interaction in favour of the intervention	Significant improvement in various HrQoL domains compared to active controls
Jung et al. [40] USA	Physical and cognitive exercise (agility boot camp with cognitive challenge, ABC-C) (I) versus education/self-management + relaxation (C) Duration: 6 weeks	Total *n* = 86; *n* = 28 included (MCI) Demographics of whole sample: 68.8 years (7.6); 67% M; H & Y Stage I–III 91%, H & Y Stage IV 9%	No cognitive criteria. Secondary analysis stratified by cognition: SCOPA-COG < 27 as MCI	Baseline; between interventions; after both intervention crossover trial	*Reported as score change: Mean (standard error)*			Balance-Improvement after exercise but not education	No significant improvement in ADLs or HrQoL for MCI subgroup. Note, measures taken in ‘off'-state
					*PDQ39* b I: −0.22 (1.39); C: −1.56 (0.96) *β* = −1.43 (CI -4.67-1.80), *p*=0.04	Not measured	MDS-UPDRS part IIb I:-0.95 (0.87); C: −0.52 (0.83) *β* = 0.26 (CI-2.01–2.54), *p*=0.8		
Giguere-Rancourt et al. [41] Canada	Goal management training (I1) versus psychoeducation with mindfulness (I2) Duration: 5 weeks.	*N* = 12 I1: 71.0 years (4.0), 83% M; H & Y 1.9 (0.6) I2: 70.0 years (5.1), 83% M; H & Y 1.7 (0.8)	PD-MCI by MDS diagnostic criteria inclusive of executive dysfunction, and MoCA 21–27. Excluded PDD	Baseline; midintervention; 1 week post; 4 weeks; 12 weeks post	*PDQ39* b I1: 31.48 (SE 5.88); I2: 20.14 (SE 5.58)^c^ Time–group interaction⁣^∗^: *F*(4,36) = 5.31, *p*=0.002, (95%CI = 15.33–25.61), *n*2 = 0.066 (medium ES in favour of I2)	Not measured	Not measured	Executive function—Improvement in both groups, group difference not significant	Significant improvement in HrQoL in psychoeducation with mindfulness group over goal management training group

Abbreviations: C = control group, EF = executive function, ES = effect size, H & Y = Hoehn & Yahr stage, tDCS = transcranial direct current stimulation, I = intervention group, M = male.

^a^Higher = better, ^b^lower = better, ^c^data obtained from authors, ^d^adjusted mean difference, adjusted for baseline outcome value.

⁣^∗^Statistically significant at *p* < 0.05.

**Table 3:** Intervention details for all studies.

Study and intervention	Target	Intervention description	Intervention adaptations for PD	Delivery and provider	Dose	Modifications/tailoring	Fidelity and engagement	Comparator details
*Randomised controlled trials*								
Kalbe et al. & Schmidt et al. [29, 30] Cognitive training: NEUROvitalis programme	Executive function, memory, attention and visuocognition	Developed by neuropsychologists. Published material with a detailed manual. Each session uses training elements: Psychoeducation (e.g., possible cognitive decline in PD, memory strategies), group tasks and activity games, individual exercises and homework. Handouts provided	Two sessions were modified considering the characteristic cognitive profile in PD: two memory sessions were replaced by sessions focussing on executive functions and visuocognition	Group-based (3–5 participants). Facilitators: Psychologists, gerontologists trained for conducting the programme	2 × 90 min/week over 6 weeks Total 12 sessions.	Two levels of severity to adapt for patients' cognitive profile	Attendance record + training diary (designed as feasibility measures) Participants in both groups attended on average 11 (range 8–12) training sessions.	Group-based (3-5 participants) physical activity training: low intensity: warm-up, stretching, flexibility, loosening or relaxation exercises, psychoeducation, homework developed by a sports scientist. Same dose.
Sousa et al. [31] Cognitive training	Cognitive function, emphasising attention and executive dysfunction	Paper and pencil tasks, practising structured exercises. Participants explored and resolved the tasks, then discussed with other participants and selected the most effective strategy. Each session targeted a particular cognitive domain. Task example: find and mark equal figures among other similar ones	Emphasised the specific areas of cognitive deficit in this population: Attention and executive dysfunction	Conducted by 2 professional cognitive experts, within a hospital neurorehabilitation programme	2 × 2 h/week, over 4 weeks Total 8 sessions	3 levels of difficulty offered	No mention	Activities of the general rehabilitation programme
Bernini et al. [32] Cognitive training plus standard physical rehabilitation: CoRe	Cognitive function (note executive functions addressed are specified in the article)	Cognitive training: Ontology-based software tool. Example exercises: find the category; unscramble the sentence. Standard physical rehabilitation: Cardiovascular warm-up, active and passive range of motion exercises, abdominal stretches, muscle strengthening, postural changes and balance and postural control exercises	—	Computer based for cognitive training	3 × 45 min/week over 4 weeks. Total 12 sessions	CoRe software tailors exercises to patients	No mention	Standard physical rehabilitation
Lawrence et al. [33] Cognitive training: SmartBrain Pro	Designed to train each cognitive domain	Standard cognitive training: Completed a predetermined programme comprising 10 activities, two activities per cognitive domain, e.g., similarities and differences. Same range of activities used for tailored cognitive training group but individualised selection	—	Interactive computer-based training programme streamed over internet for use on home computer or device provided by researcher	3 × 45 min/week over 4 weeks. Total 12 sessions	Performance monitored by the programme and difficultly levels adjusted. Tailored group: Exercises were individualised to their baseline neuropsychological test results	No mention	6-arm study: Cognitive training, standard and tailored, each with and without tDCS; tDCS alone; and control (no intervention)
Folkerts et al. [34] Cognitive stimulation: NEUROvitalis senseful	Executive function, memory, spatial and social cognition; fine motor function; and sensory perception (auditory, tactile, olfactory and visual)	Manualised cognitive stimulation—each session includes 25 min of cognitive exercises (e.g., category memory ga), 10 min of fine motor skills training (e.g., knot game) and 15 min of sensory stimulation (e.g., describing and naming sounds)	Adapted according to typical PD deficits: added more exercises targeting executive, visual–spatial and motor functions	Group-based (3-5 participants). Delivered in PD care unit by Dutch psychologists, trained by one of the programme developers	2 × 60 min/week for 8 weeks. Total 16 sessions	—	Participation recorded: Attended between 11 and 16 CS sessions (participation score: 92.7%)	Usual care (including a variety of nonpharmacological interventions such as sports, music and arts, open to all participants voluntarily)
Leroi et al. [35] Cognitive stimulation	Neurobiological and psychosocial aspects. Intervention is based on principles of errorless learning and validation	Cognitive stimulation: Engaging psychosocial intervention with cognitively stimulating activities and discussions. The manual includes 60 topics within 9 themes, with cognitive stimulating activities within each, e.g., discussion topics, word association games and creative tasks. Paper based indexed manual, in large accessible print	Iteratively developed by researchers, clinicians and PPI. Parkinson's-adapted, e.g. removal of discrete levels of task complexity, removal of images that were potentially hallucinogenic or lacked clarity, and updating of the content	Delivered by care partners at home. 2 h protocolised training for care partners. Researcher provided additional training and telephone support as needed, based on a skills checklist	2–3x 30 min/week for 12 weeks. Total 24-36 sessions	Activities varied in theme and complexity—tailored to the individual	Training checklist, training evaluation form, therapy skills self-assessment, and diary entries. > 90% undertook discrete sessions > 20 min duration, but average number of sessions completed was lower than recommended dose	Usual care
Hindle et al. [37] Cognitive rehabilitation: CORD-PD	Goal-based, targeting difficulties relating to orientation, planning, the retention of learned information and recall	Involved goal setting then teach compensatory strategies and/or restorative approaches to circumnavigate cognitive difficulties. Strategies for managing practical situations and cognitive difficulties as well as anxiety symptoms are introduced across the sessions	—	Individual sessions with an occupational therapist experienced in neurorehabilitation. Carers invited	1 × 1 h/week for 8 weeks. Total 8 sessions	Individually tailored to participants goals	Therapist's adherence to the treatment protocols will be monitored through therapy logs and regular supervision sessions. Completion of 6/8 sessions considered sufficient adherence: 80% met this in intervention group, 90% in active control	Active control: Relaxation therapy (RT)—muscle relaxation and breathing exercises Inactive control: Usual care
Vlagsma et al. [38] Cognitive rehabilitation & strategy training: eSET	Executive function, to improve or stabilise independence and QoL	Teaches strategies for everyday life to compensate for impairments. 3 modules: Information and awareness (3 sessions); goal setting and planning (6); Initiative, execution and regulation (5). Some included relatives. 3 goals set in third session. Changes made at home	Adapted for PD by reducing number of sessions and number of exercises per session	Individual treatment delivered by neuropsychologists	14 × 1 hr sessions (once or if possible twice a week) plus homework assignments. Total 14 sessions	Not specified	No mention	‘CogniPlus' 14 × 1 hr sessions, computer training programme addressing attention and working memory, technical supported by psychology test assistant, also asked to set 3 goals in the third session. No practice possible outside of the sessions.
Reuter et al. [39] Multimodal cognitive rehabilitation: Cognitive training plus transfer training plus psychomotor and endurance training	All cognitive domains; coping; inhibitory control; motor skills; coordination, strength, speed, perception and orientation; body perception	Cognitive tasks, e.g., object assembly; story telling. Transfer: Practice and strategies for daily tasks, e.g., prepare a meal, pay a bill. Psychomotor and endurance games and tasks, e.g., dual tasking; finding hidden items, + aerobic exercise (indoor and outdoor if possible). Given instructions for continuing training at home. Caregivers given PD and caregiving education	Focussed on PD-specific impairments	Individual 1:1 treatment, delivered by physiotherapists, occupational therapists and neuropsychologists Some training exercises were on a computer. Caregiver education by a specialist nurse, physiotherapist and psychologist	Cognitive training: 4 × 1 h/week. Transfer training: 3 × 90 min/week. Motor training: 1 h x 10–12 over 4 weeks. Home phase: Instructed to do 45 min x 3 cognitive, x 2 transfer and x 2 motor training sessions/week	Cognitive training: Individually tailored to baseline test results and difficulty adapted to performance. Transfer training: Composed according to baseline test results. Motor training: Based on individual capabilities and needs	All participants completed 14 cognitive, 10 transfer and 10 motor training sessions. Participants and caregivers kept a diary. Home phase: 90% pursued the training at home. Control 1: 60% did as advised. Control 2: 60% for transfer tasks; 75% for cognitive	Control 1: just cognitive training; Control 2: control training + transfer training. Both had additional relaxation training and occupational training (without transfer component) to bring all groups to the same total dose
Jung et al. [40] Physical and cognitive exercise: Agility boot camp with cognitive challenge, ABC-C	Executive function, attention and mobility	Agility boot camp with cognitive challenge (ABC-C): Simultaneous physical and cognitive tasks, run as circuits: gait training, PWR! moves, agility course, lunges, boxing and adapted Tai Chi. Each station 10–20 min, short rest between	Based on a framework set out specifically for PD	Group class (3–6/class) led by certified exercise trainers experienced in PD + 1–2 research assistants to spot participants	3 × 80 min/week for 6 weeks. Total 18 sessions	Systematic progression of difficulty for each participant	‘Compliance' recorded by the trainer at each session and coded progression. Moderately high compliance seen (> 65%)	Education/self-management (same group, trainer) once/week (80 min) + relaxation videos for home (6 × 30 min/week), to match the total ‘dose'
Giguere-Rancourt et al. [41] I1: Goal management Training.I2: PSYCH-mind	Strategy-based targeting executive function	Exercises include self-management strategies, self-monitoring, cognitive training techniques, psychoeducation about cognition Included 20–30 min mindfulness exercises per session (4 exercises: Mental visualisation, body scan, long breathing, short breathing). Exercises to do at home: mindfulness and metacognitive reflection. Workbook provided	Reduced session duration and number, removed repetition, removed tasks requiring dexterity, PD-specific information added, individual iPad rather than group presentation	Delivered at home by study investigator, in the presence of caregiver, individually using an iPad. Workbook provided	5 × 60–90 min sessions over 5 weeks, plus homework. Total 5 sessions	Not specified	Feasibility measures for implementation: Degree of execution and success or failure of execution All participants were able to execute the exercises and reflections appropriately. 1 participant in I2 fell asleep during. In I1, 1 participant did more than recommended at home, 1 did less, others as recommended. 3 in I2 did mindfulness exercises at home (had not been asked to)	Comparison between interventions as detailed, no control arm
	Understanding of their condition	Psychoeducation with mindfulness. 5 modules: Brain and motor symptoms; autonomic symptoms; psychological symptoms; brain and cognition; cognitive impairments in PD. Each session is composed of 40–60 min information plus 20–30 min mindfulness exercises (four exercises as above)	Designed specifically for the study, with patients and caregivers	Delivered at home, by study investigator, in the presence of caregiver. Information book provided	5 × 60–90 min sessions over 5 weeks Total 5 sessions	Not specified		
*Nonrandomised controlled trials*								
Disbrow et al. [42] ‘Neurorehabilitation' (arguably cognitive training rather than rehabilitation)	Simple motor performance and motor-related executive function, specifically internally represented sequencing and switching	Series of cognitive tasks (sequencing) presented on ‘Neurobehavioural Systems' software. 15-min training for using the computer and software at the start. Given 12 pairs of practice trials (different cueing types). Given training log and contact information for the trainer. Each session had 8 sections of 5 min with flexible breaks. Schedule of feedback provided	Tasks chosen due to research question regarding cognition in PD but no description of intervention being tailored to PD	Computer-based: Standardised computers set up in participants home	5 × 40 min sessions/week over 12–14 days. Total 10 sessions	Level of external cueing varied. Task difficulty adjusted according to reaction time and errors	Training log described. Adherence not reported	Pre-/postintervention. Non-PD control group
Formica et al. [43] Computer-assisted rehabilitation environment (CAREN)	Primarily walking, but also attention, memory, language, executive function, spatial cognition and perceptive abilities. Also motivation, strategy and (unlearn-) poor habits	Virtual reality rehabilitation: Operator generates physical, visual and cognitive perturbations requiring the participant to make dynamic responses. Included walking, balance and coordination. Increased difficulty every 5 sessions	No tailoring to PD as such, but selected scenarios were relevant to PD impairments	Delivered via a virtual reality device, supported by a physical therapist	50 min sessions x 2 in first week then x 3/week for 2 months. Total 24 sessions	None reported	No mention	Pre-/postintervention, no control
Gandy et al. [44] Psychological intervention: Wellbeing Neuro Course	Psychological skills to manage mental health and functional abilities	Psychological intervention integrating CBT with compensatory cognitive rehabilitation therapy. Provides information and teaches practical skills for managing the impact of condition. 6 core lessons (each ∼ 60-slide slideshow, self-paced) + 6 summaries which provide worksheets to learn and practice core skills. Additional resources provided	Tailored to neurological disorders but not PD	Internet-delivered. Course materials released systematically over 10 weeks + email/telephone contact from a clinical psychologist encouraged to complete it with a supporter, e.g., caregiver/friend	Self-paced course over 10 weeks. Weekly remote contact from clinical psychologist: ∼ 10–15 min, more if needed. Total 10 sessions	Self-paced so can vary	Adherence and acceptability measures. For participants with PD: Postintervention 82% had completed all 6 lessons, 88% by 3 months of follow-up	Pre-/postintervention, no control

Abbreviations: CBT, cognitive behavioural therapy; PPI, public and patient involvement; tDCS: transcranial direct current stimulation.

**Table 4:** GRADE summary of evidence table.

Nonpharmacological interventions for people with Parkinson's and cognitive impairment				
*Population*: People with idiopathic Parkinson's disease and cognitive impairment				
*Setting*: Any				
*Intervention*: Nonpharmacological interventions (only cognitive interventions represented by meta-analysis)				
*Comparison*: Control arm (varied comparators: Usual care; physical activity/rehabilitation; cognitive training)				
*Outcomes* Immediately postintervention (up to 2 weeks post end of intervention)	*Comparative risks: Standardised mean difference (95% Cis)*	*No. of participants (studies)*	*Quality of the evidence (Grade)*	*Comments*
*Health-related quality of life* PDQ-39, PDQ-8, Qualidem	−0.20 [−0.57, 0.18]	235 (7^∗^)	⊕⃝⃝⃝ Very lowa^,^^b^^,^^c^^,^^d^	Negative values indicate improved health-related quality of life (signs reversed for Qualidem to match direction of effect).
*Function/Activities of daily Living* Bayer ADLs, BAFQ, UPDRS Part II, pill questionnaire	0.14 [−0.23, 0.51]	200 (5^∗^)	⊕⃝⃝⃝ Very lowa^,^^b^^,^^c^^,^^d^	Positive values indicate worsened function.
*Wellbeing* Relatives stress scale, brief resilience scale, generalised efficacy scale	See comment	105 (2)	⊕⃝⃝⃝ Very low	Outcomes address different aspects of wellbeing rather than a unified construct and so not considered suitable for meta-analysis.

⁣^∗^One study is divided into two independent intervention arms, with the control group halved for inclusion in the meta-analysis.

^a^Downgraded by one point due to the risk of bias of included studies: risk about randomisation in two trials, risk from crossover (carryover) in one, risk about outcome measurement due to lack of blinding (expected due to nature of interventions) in 5 of the 7 trials included in HrQoL analysis, and 2 of the 4 for function analysis.

^b^Downgraded by one point due to inconsistency: moderate heterogeneity. Some can be accounted for by subgroup analysis by intervention type, comparator type and cognitive diagnosis of participants.

^c^Downgraded by one point due to imprecision: confidence intervals cross two possible interpretations (benefit and harm).

^d^Downgraded by one point due to detection of publication bias on funnel plot where possible and extrapolated when too few studies for funnel plot.

## Data Availability

All relevant data are included in the manuscript and supporting information.

## Supporting Information

Full search strategy. Table of cognitive function of participants across all studies. Full quality assessments. Forest plots for 3- to 6-month follow-up. Forest plots for subgroup analyses.
