# Supplementary material for: Effectiveness and Feasibility of Nonpharmacological Interventions for People With Parkinson's Disease and Cognitive Impairment on Patient-Centred Outcomes: Systematic Review and Meta-Analysis
Source: Parkinsons Dis. 2024 Nov 18;2024:3654652. doi: 10.1155/2024/3654652 (PMC11588407; doi:10.1155/2024/3654652)
Supplement: Supporting Information — Additional supporting information can be found online in the Supporting Information section. [file 3654652.f1.docx]

**Supplementary Materials**

[1. Full search strategy](#search)

[2. Table of cognitive function of participants across all studies](#cognition)

[3. Full Quality Assessments](#quality)

[4. Forest plots for 3-6month follow-up](#followup)

[5. Forest plots for subgroup analyses](#subgroup)

**1. Full Search Strategy**

MEDLINE

1 parkinsonian disorders/ or parkinson disease/

2 parkinson*.mp. [mp=title, abstract, original title, name of substance word, subject heading word, floating sub-heading word, keyword heading word, organism supplementary concept word, protocol supplementary concept word, rare disease supplementary concept word, unique identifier, synonyms]

3 1 or 2

4 Dementia/

5 cognition disorders/ or auditory perceptual disorders/ or cognitive dysfunction/

6 (cognit* adj3 (decline or dysfunction* or impair* or disorder* or deteriorat*)).mp. [mp=title, abstract, original title, name of substance word, subject heading word, floating sub-heading word, keyword heading word, organism supplementary concept word, protocol supplementary concept word, rare disease supplementary concept word, unique identifier, synonyms]

7 (neurocognit* adj3 (decline or dysfunction* or impair* or disorder* or deteriorat*)).mp. [mp=title, abstract, original title, name of substance word, subject heading word, floating sub-heading word, keyword heading word, organism supplementary concept word, protocol supplementary concept word, rare disease supplementary concept word, unique identifier, synonyms]

8 (mental* adj3 (decline or dysfunction* or impair* or disorder* or deteriorat*)).mp. [mp=title, abstract, original title, name of substance word, subject heading word, floating sub-heading word, keyword heading word, organism supplementary concept word, protocol supplementary concept word, rare disease supplementary concept word, unique identifier, synonyms]

9 4 or 5 or 6 or 7 or 8

10 "quality of life"/

11 "Value of Life"/

12 "Activities of Daily Living"/

13 quality of life.mp. [mp=title, abstract, original title, name of substance word, subject heading word, floating sub-heading word, keyword heading word, organism supplementary concept word, protocol supplementary concept word, rare disease supplementary concept word, unique identifier, synonyms]

14 (wellbeing or well being).mp. [mp=title, abstract, original title, name of substance word, subject heading word, floating sub-heading word, keyword heading word, organism supplementary concept word, protocol supplementary concept word, rare disease supplementary concept word, unique identifier, synonyms]

15 (activit* adj3 (life or living)).mp. [mp=title, abstract, original title, name of substance word, subject heading word, floating sub-heading word, keyword heading word, organism supplementary concept word, protocol supplementary concept word, rare disease supplementary concept word, unique identifier, synonyms]

16 (function* adj3 (abilit* or disabilit* or decline or impair* or deteriorat*)).mp. [mp=title, abstract, original title, name of substance word, subject heading word, floating sub-heading word, keyword heading word, organism supplementary concept word, protocol supplementary concept word, rare disease supplementary concept word, unique identifier, synonyms]

17 10 or 11 or 12 or 13 or 14 or 15 or 16

18 exp Social Support/

19 (support* or information or advis* or advice or intervention* or therap* or program* or train* or educat*).mp. [mp=title, abstract, original title, name of substance word, subject heading word, floating sub-heading word, keyword heading word, organism supplementary concept word, protocol supplementary concept word, rare disease supplementary concept word, unique identifier, synonyms]

20 health education/ or patient education as topic/

21 exp Psychotherapy/

22 Physical Therapy Modalities/

23 Occupational Therapy/

24 Speech Therapy/

25 18 or 19 or 20 or 21 or 22 or 23 or 24

26 3 and 9 and 17 and 25

Embase

1 parkinsonism/

2 Parkinson disease/

3 parkinson*.mp. [mp=title, abstract, heading word, drug trade name, original title, device manufacturer, drug manufacturer, device trade name, keyword, floating subheading word, candidate term word]

4 1 or 2 or 3

5 dementia/

6 mild cognitive impairment/

7 cognitive defect/

8 (cognit* adj3 (decline or dysfunction* or impair* or disorder* or deteriorat*)).mp. [mp=title, abstract, heading word, drug trade name, original title, device manufacturer, drug manufacturer, device trade name, keyword, floating subheading word, candidate term word]

9 (neurocognit* adj3 (decline or dysfunction* or impair* or disorder* or deteriorat*)).mp. [mp=title, abstract, heading word, drug trade name, original title, device manufacturer, drug manufacturer, device trade name, keyword, floating subheading word, candidate term word]

10 (mental* adj3 (decline or dysfunction* or impair* or disorder* or deteriorat*)).mp. [mp=title, abstract, heading word, drug trade name, original title, device manufacturer, drug manufacturer, device trade name, keyword, floating subheading word, candidate term word]

11 5 or 6 or 7 or 8 or 9 or 10

12 quality of life.mp. [mp=title, abstract, heading word, drug trade name, original title, device manufacturer, drug manufacturer, device trade name, keyword, floating subheading word, candidate term word]

13 (wellbeing or well being).mp. [mp=title, abstract, heading word, drug trade name, original title, device manufacturer, drug manufacturer, device trade name, keyword, floating subheading word, candidate term word]

14 (activit* adj3 (life or living)).mp. [mp=title, abstract, heading word, drug trade name, original title, device manufacturer, drug manufacturer, device trade name, keyword, floating subheading word, candidate term word]

15 (function* adj3 (abilit* or disabilit* or decline or impair* or deteriorat*)).mp. [mp=title, abstract, heading word, drug trade name, original title, device manufacturer, drug manufacturer, device trade name, keyword, floating subheading word, candidate term word]

16 exp "quality of life"/

17 wellbeing/ or physical well-being/ or psychological well-being/

18 daily life activity/

19 functional status/

20 exp functional status assessment/

21 12 or 13 or 14 or 15 or 16 or 17 or 18 or 19 or 20

22 social support/

23 (support* or information or advis* or advice or intervention* or therap* or program* or train* or educat*).mp. [mp=title, abstract, heading word, drug trade name, original title, device manufacturer, drug manufacturer, device trade name, keyword, floating subheading word, candidate term word]

24 patient information/

25 health education/ or patient education/ or psychoeducation/

26 exp psychotherapy/

27 physiotherapy/ or chest wall oscillation/ or home physiotherapy/ or joint mobilization/

28 occupational therapy/

29 exp "speech and language rehabilitation"/

30 social care/ or caregiver support/ or psychosocial care/

31 22 or 23 or 24 or 25 or 26 or 27 or 28 or 29 or 30

32 4 and 11 and 21 and 31

CINAHL

S1 (MH "Parkinson Disease") OR (MH "Parkinsonian Disorders")

S2 parkinson*

S3 S1 OR S2

S4 (MH "Cognition Disorders+") OR (MH "Dementia")

S5 cognit* N3 (decline or dysfunction* or impair* or disorder* or deteriorat*)

S6 neurocognit* N3 (decline or dysfunction* or impair* or disorder* or deteriorat*)

S7 mental* N3 (decline or dysfunction* or impair* or disorder* or deteriorat*)

S8 (MH "Quality of Life+")

S9 (MH "Psychological Well-Being")

S10 (MH "Attitude to Life")

S11 (MH "Personal Satisfaction+")

S12 (MH "Activities of Daily Living+")

S13 (MH "Caregiver Burden")

S14 "quality of life"

S15 wellbeing

S16 (activit* N3 (life or living))

S17 (function* N3 (abilit* or disabilit* or decline or impair* or deteriorat*))

S18 S4 OR S5 OR S6 OR S7

S19 S8 OR S9 OR S10 OR S11 OR S12 OR S13 OR S14 OR S15 OR S16 OR S17

S20 (support* or information or advis* or advice or intervention* or therap* or program* or train* or educat*)

S21 (MH "Health Education") OR (MH "Patient Education")

S22 (MH "Physical Therapy+") OR (MH "Occupational Therapy+")

S23 (MH "Psychotherapy+")

S24 (MH "Rehabilitation, Speech and Language+"))

S25 (MH "Support, Psychosocial+")

S26 S20 OR S21 OR S22 OR S23 OR S24 OR S25

S27 S3 AND S18 AND S19 AND S26

APA PsycInfo

1 parkinson's disease/

2 parkinson*.mp. [mp=title, abstract, heading word, table of contents, key concepts, original title, tests & measures, mesh]

3 1 or 2

4 dementia/ or cognitive impairment/

5 (cognit* adj3 (decline or dysfunction* or impair* or disorder* or deteriorat*)).mp. [mp=title, abstract, heading word, table of contents, key concepts, original title, tests & measures, mesh]

6 (neurocognit* adj3 (decline or dysfunction* or impair* or disorder* or deteriorat*)).mp. [mp=title, abstract, heading word, table of contents, key concepts, original title, tests & measures, mesh]

7 (mental* adj3 (decline or dysfunction* or impair* or disorder* or deteriorat*)).mp. [mp=title, abstract, heading word, table of contents, key concepts, original title, tests & measures, mesh]

8 4 or 5 or 6 or 7

9 "Quality of Life"/

10 well being/ or mental health/

11 "activities of daily living"/ or daily activities/

12 life satisfaction/

13 quality of life.mp. [mp=title, abstract, heading word, table of contents, key concepts, original title, tests & measures, mesh]

14 (wellbeing or well being).mp. [mp=title, abstract, heading word, table of contents, key concepts, original title, tests & measures, mesh]

15 (activit* adj3 (life or living)).mp. [mp=title, abstract, heading word, table of contents, key concepts, original title, tests & measures, mesh]

16 (function* adj3 (abilit* or disabilit* or decline or impair* or deteriorat*)).mp. [mp=title, abstract, heading word, table of contents, key concepts, original title, tests & measures, mesh]

17 9 or 10 or 11 or 12 or 13 or 14 or 15 or 16

18 exp Social Support/

19 (support* or information or advis* or advice or intervention* or therap* or program* or train* or educat*).mp. [mp=title, abstract, heading word, table of contents, key concepts, original title, tests & measures, mesh]

20. health education/ or psychoeducation/

21 exp psychotherapy/

22 physical therapy/ or occupational therapy/

23 speech therapy/ or language therapy/

24 18 or 19 or 20 or 21 or 22 or 23

25 3 and 8 and 17 and 24

Web of science:

1 .TS=(parkinson*)

2. TS=((cognit* NEAR/3 (decline or dysfunction* or impair* or disorder* or deteriorat*)) OR (neurocognit* NEAR/3 (decline or dysfunction* or impair* or disorder* or deteriorat*)) OR (mental* NEAR/3 (decline or dysfunction* or impair* or disorder* or deteriorat*)))

3. TS=("quality of life" OR wellbeing or "well being" OR (activit* NEAR/3 (life or living)) OR (function* NEAR/3 (abilit* or disabilit* or decline or impair* or deteriorat*)))

4. TS=(support* OR information OR advis* OR advice OR intervention* OR therap* OR program* OR train* OR educat*)

5. #4 AND #3 AND #2 AND #1

**2. Cognitive function of participants across all studies**

| **Study** | **Cognitive Inclusion Criteria** | **Global Cognition at Baseline Assessment**  *Mean (sd) unless otherwise stated* | | |
| --- | --- | --- | --- | --- |
|  |  | **Measure** | **Intervention Group** | **Control Group** |
| **Kalbe et al (2020) & Schmidt et al (2021)** | MDS Level II Criteria: PD-MCI and subjective cognitive impairment and/or MoCA<26. Excluded PDD. | MoCA | Kalbe sample-  25 (16-28)  Schmidt sample-  25.0 (2.22) | Kalbe sample-  25 (15-30)  Schmidt sample- 24.23 (3.15) |
| **Sousa et al**  **(2021)** | MDS Level II Criteria: PD-MCI | ACE-III | 87.5 (6.6) | 87.1 (6.9) |
| **Bernini et al (2019)** | MDS Level II Criteria: PD-MCI single or multiple domain but must include executive domain on | MMSE | 25.32 (2.26) | 25.35 (2.68) |
|  |  | MoCA | 20.82 (3.34) | 19.17 (2.49) |
| **Lawrence et al (2018)** | MDS Level II Criteria: PD-MCI; cognitive deficits that did not interfere with functional independence. | MMSE | sCT: 26.95 (2.09)  tCT: 25.49 (2.44) | 24.38 (2.07) |
| **Folkerts et al (2018)** | Residents of PDD-specific long term care unit; MMSE 10-25 | MMSE | 17.50 (5.75) | 18.17 (5.35) |
| **Leroi et al (2019)** | MDS Level 1 Criteria: PD-MCI; PDD (probable or possible); DLB (probable or possible) | ACE-III | 68.69 (14.73) | 63.78 (15.15) |
|  |  | MoCA  *Median (IQR)* | 17.5 (15-21.5) | 19 (15-22) |
| **Hindle et al (2018)** | MDS Criteria: PDD or DLB diagnosed according to and ACE-III ≤82 | ACE-III | 71.6 (6.74) | Relaxation therapy: 71.9 (7.19)  Usual care: 70.22 (9.38) |
| **Vlagsma et al (2020)** | Problems in executive function in everyday life: semi-structured interview and/or ≥18 on DEX + impairment on EF tests | SCOPA-COG | 28.29(4.76) | 28.79 (4.70) |
| **Reuter et al (2012)** | PD-MCI: cognitive decline symptoms,  preferably corroborated, end cognitive abnormalities which cannot  be simply attributed to age but with minimal  effect on daily function and no dementia. | SCOPA-COG | 31.83 (3.21) | C1: 29.07 (3.8)  C2: 29.68 (2.87) |
| **Jung et al**  **(2020)** | *No cognitive criteria.*  Secondary analysis stratified by cognition: SCOPA-COG<27 as MCI subgroup | SCOPA-COG (Whole group, not provided for MCI subgroup) | 28.1 (4.9) | |
| **Giguere-Rancourt et al (2022)** | MDS Criteria: PD-MCI inclusive of executive dysfunction, and MoCA 21-27. Excluded PDD. | MoCA | GMT: 24.00 (2.19)  PsychMIND: 23.83 (2.32) |  |
| **Disbrow et al (2012)** | *Not* specifically targeting cognitive impairment; categorised after assessment. Excluded if immediate memory impairment or dementia. Impaired PD subgroup included. | *Global cognition not reported*. | | |
|  |  | Digit Span | 15.5 (2.2) | NA |
|  |  | Trails Task B-A | 79.8 (38.7) | NA |
| **Formica et al (2023)** | “Selected for” Moderate to mild cognitive impairment (MoCA>15) | MoCA  *Median (IQR)* | 24 (20.5-26.5) | NA |
| **Gandy et al (2020)** | Experiencing cognitive difficulties impacting day-to-day activities and QoL. Excluded serious cognitive impairment indicative of dementia. | Not reported | | |

Cognitive assessments & maximum scores: MMSE=Mini Mental State Examination /30; MoCA =Montreal Cognitive Assessment /30; ACE-III=Addenbrookes Cognitive Assessment /100, RBANS= Repeatable Battery for the Assessment of Neuropsychological Status /160, SCOPA-COG= Scales for Outcomes in Parkinson's disease-Cognition /43.

Abbreviations: sCT: standard cognitive training, tCT: tailored cognitive training, GMT: Goal Management Training; DEX: dysexecutive questionnaire

**3. Full Quality Assessments**

1. **GRADE full assessment table**

|  | **RoB** | **Inconsistency** | **Indirectness** | **Imprecision** | **Publication Bias** | **Overall quality of evidence** |
| --- | --- | --- | --- | --- | --- | --- |
| **HrQoL post-intervention** | 2 low, 2 some concern, 3 high 🡪 -1 | I^2^= 48.21%, test of Q=0, p=0.07 🡪 -1  Some explained by subgroup analysis but not convincingly to make distinctive recommendation | For cognitive interventions, no concerns, unable to generalise to other interventions | Wide confidence intervals, crossing two possible interpretations 🡪 -1 | Detected on funnel plot 🡪 -1 | Very low |
| **Function post-intervention** | 2 low, 1 some concern, 2 high 🡪 -1 | I^2^=36.08%, test of Q=0, p=0.15 🡪 -1  Some explained by subgroup analysis but not convincingly to make distinctive recommendation | For cognitive interventions, no concerns, unable to generalise to other interventions | Wide confidence intervals, crossing two possible interpretations 🡪 -1 | Unlikely from funnel plot | Very low |
| **Wellbeing post-intervention** | 2 some concern 🡪 -1 | Only 2 studies. Not synthesised numerically due to different measures, but one significant, the other not. | No direct measures of wellbeing, measures are of related constructs that we consider to contribute to wellbeing 🡪 -1 | Small sample size 🡪 -1 | Too few studies, but likely given the results for other outcomes 🡪 -1 | Very low |
| **HrQoL at follow-up (3-6months)** | 2 low, 1 some concern, 2 high of bias 🡪 -1 | I^2^=52.37%, test of Q=0, p=0.07 🡪 -1  Some explained by subgroup analysis but not convincingly to make distinctive recommendation | For cognitive interventions, no concerns, unable to generalise to other interventions | Wide confidence intervals, crossing two possible interpretations 🡪 -1 | Detected on funnel plot 🡪 -1 | Very low |
| **Function at follow-up (3-6months)** | 2 low, 1 some concern, 1 high of bias 🡪 -1 | I^2^=0%, test of Q=0, p=0.52 🡪  No change | For cognitive interventions, no concerns, unable to generalise to other interventions | Wide confidence intervals, crossing two possible interpretations 🡪 -1 | Detected on funnel plot 🡪 -1 | Very low |
| **Wellbeing at follow-up (3-6months)**  (1 study) | 1 some concern 🡪 -1 | Only one small study, no inconsistency | No direct measures of wellbeing, self-efficacy considered since it may contribute to wellbeing 🡪 -1 | Small sample size 🡪 -1 | Too few studies, but likely given the results for other outcomes 🡪 -1 | Very low |

1. **Risk of bias graph: review authors' judgements about each risk of bias item presented as percentages across all included RCTs**


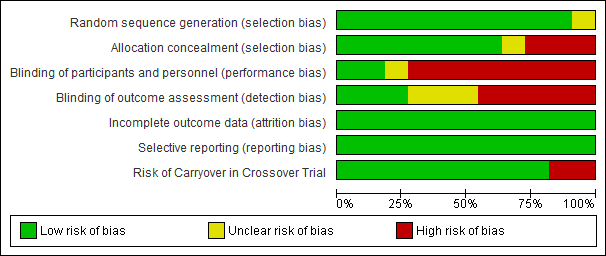


1. **Risk of Bias in Randomised Controlled Trials**

**
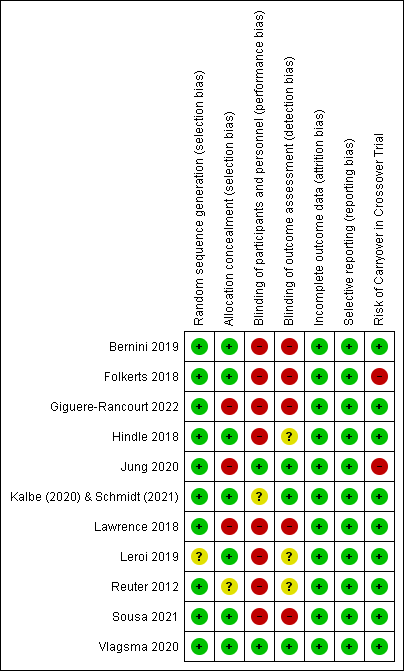
**

| 1. **Risk of Bias in Randomised Controlled Trials: Cochrane Risk of Bias 2 (RoB2) tool** | | | | | | | | | | | | |
| --- | --- | --- | --- | --- | --- | --- | --- | --- | --- | --- | --- | --- |
| **Domains** | **Signalling questions** | **Kalbe 2020 & Schmidt 2021** | **Sousa**  **2021** | **Bernini 2019** | **Lawrence 2018** | **Folkerts 2018** | **Leroi 2019** | **Hindle 2018** | **Jung**  **2020** | **Vlagsma 2020** | **Reuter 2012** | **Giguere-Rancourt 2022** |
| 1.Randomization process: | 1.1Was the allocation sequence random? | Y | Y | Y | Y | Y | Y | Y | PY | Y | Y | PY |
|  | 1.2Was the allocation sequence concealed until participants were enrolled and assigned to interventions? | PY | PY | PY | PN | Y | PY | Y | PN | PY | NI | PN |
|  | 1.3Did baseline differences between intervention groups suggest a problem with the randomization process? | PN | N | N | N | N | PY | N | PN | N | N | PN |
|  | Risk-of-bias judgement | Low | Low | Low | High^ | Low | Some concern | Low | High^ | Low | Some concern | High^ |
| S. Risk of bias arising from period and carryover effects  (crossover trials only) | S.1 Was the number of participants allocated to each of the two sequences equal or nearly equal? | NA | NA | NA | NA | Y | NA | NA | Y | NA | NA | NA |
|  | S.2 If N/PN/NI to S.1: Were period effects accounted for in the analysis? |  |  |  |  | NA |  |  | NA |  |  |  |
|  | S.3 Was there sufficient time for any carryover effects to have disappeared before outcome assessment in the second period? |  |  |  |  | PN |  |  | PN |  |  |  |
|  | Risk-of-bias judgement |  |  |  |  | High |  |  | High |  |  |  |
| 2. Deviation from Intended intervention:  (Intention-to treat-) | 2.1. Were participants aware of their assigned intervention during the trial? | PN | Y | Y | Y | Y | Y | Y | PN | Y | PY | Y |
|  | 2.2. Were carers and people delivering the interventions aware of participants' assigned intervention during the trial? | PY | Y | Y | Y | Y | Y | Y | Y | Y | PY | Y |
|  | 2.3. If Y/PY/NI to 2.1 or 2.2: Were there deviations from the intended intervention that arose because of the trial context? | PN | PN | N | N | N | N | PN | PN | N | PN | N |
|  | 2.4 If Y/PY to 2.3: Were these deviations likely to have affected the outcome? | NA | NA | NA | NA | NA | NA | NA | NA | NA | NA | NA |
|  | 2.5. If Y/PY/NI to 2.4: Were these deviations from intended intervention balanced between groups? | NA | NA | NA | NA | NA | NA | NA | NA | NA | NA | NA |
|  | 2.6 Was an appropriate analysis used to estimate the effect of assignment to intervention? | Y | PY | Y | Y | PY | PY | Y | PY | PY | PY | PY |
|  | 2.7 If N/PN/NI to 2.6: Was there potential for a substantial impact (on the result) of the failure to analyse participants in the group to which they were randomized? | NA | NA | NA | NA | NA | NA | NA | NA | NA | NA | NA |
|  | Risk-of-bias judgement | Low | Low | Low | Low | Low | Low | Low | Low | Low | Low | Low |
| 3.Missing outcome data | 3.1 Were data for this outcome available for all, or nearly all, participants randomized? | Y | PY | PN | PY | PY | PN | PY | Y | Y | PY | Y |
|  | 3.2 If N/PN/NI to 3.1: Is there evidence that the result was not biased by missing outcome data? | NA | NA | PN | NA | NA | Y | NA | NA | NA | NA | NA |
|  | 3.3 If N/PN to 3.2: Could missingness in the outcome depend on its true value? | NA | NA | PN | NA | NA | NA | NA | NA | NA | NA | NA |
|  | 3.4 If Y/PY/NI to 3.3: Is it likely that missingness in the outcome depended on its true value? | NA | NA | N/A | NA | NA | NA | NA | NA | NA | NA | NA |
|  | Risk-of-bias judgement | Low | Low | Low | Low | Low | Low | Low | Low | Low | Low | Low |
| 4.Measurement of Outcome: | 4.1 Was the method of measuring the outcome inappropriate? | PN | PN | N | N | PN | N | N | PN | N | N | PN |
|  | 4.2 Could measurement or ascertainment of the outcome have differed between intervention groups? | PN | PN | N | N | N | N | N | PN | N | PN | PN |
|  | 4.3 If N/PN/NI to 4.1 and 4.2: Were outcome assessors aware of the intervention received by study participants? | PN | PY | Y | Y | Y | PY | PY | N | PN | PY | PY |
|  | 4.4 If Y/PY/NI to 4.3: Could assessment of the outcome have been influenced by knowledge of intervention received? | NA | PY | PY | PY | Y | PY | PY | NA | NA | PY | PY |
|  | 4.5 If Y/PY/NI to 4.4: Is it likely that assessment of the outcome was influenced by knowledge of intervention received? | NA | PY | PY | PY | PY | PN | PN | NA | NA | PN | PY |
|  | Risk-of-bias judgement | Low | High | High | High | High | Some concern | Some concern | Low | Low | Some concern | High |
| 5. Selection of reported result: | 5.1 Were the data that produced this result analysed in accordance with a pre-specified analysis plan that was finalized before unblinded outcome data were available for analysis?  Is the numerical result being assessed likely to have been selected, on the basis of the results, from. | PY | Y | Y | Y | Y | Y | Y | PY | PY | PY | PY |
|  | 5.2. ... multiple eligible outcome measurements (e.g. scales, definitions, time points) within the outcome domain? | PN | PN | PN | PN | PN | N | PN | PN | PN | PN | PN |
|  | 5.3 ... multiple eligible analyses of the data? | PN | PN | PN | PN | PN | N | PN | PN | PN | PN | PN |
|  | 5.4 (cross-over trials only) | NA | NA | NA | NA | PN | NA | NA | PN | NA | NA | NA |
|  | Risk-of-bias judgement | Low | Low | Low | Low | Low | Low | Low | Low | Low | Low | Low |
| **Overall risk of bias:** |  | **Low** | **High** | **High** | **High** | **High** | **Some concern** | **Some concern** | **High** | **Low** | **Some concern** | **High** |

^Due to block randomisation without variation in block size so potentially predictable allocations. Note assessment was conducted for HrQoL, function and wellbeing outcomes separately but the same judgments were come to.

1. **Non-Randomised Studies of Interventions – Cochrane ROBINS-I tool**

| **Signalling questions** | **Disbrow 2012** | **Formica 2023** | **Gandy 2020** |
| --- | --- | --- | --- |
| **Bias due to confounding** | | | |
| 1.1 Is there potential for confounding of the effect of intervention in this study?  **If N/PN to 1.1:** the study can be considered to be at low risk of bias due to confounding and no further signalling questions need be considered | Y | Y | PY |
| **If Y/PY to 1.1**: determine whether there is a need to assess time-varying confounding: |  |  |  |
| 1.2. Was the analysis based on splitting participants’ follow up time according to intervention received?  **If N/PN**, answer questions relating to baseline confounding (1.4 to 1.6)  **If Y/PY**, go to question 1.3. | N | N | N |
| 1.3. Were intervention discontinuations or switches likely to be related to factors that are prognostic for the outcome?  **If N/PN**, answer questions relating to baseline confounding (1.4 to 1.6)  **If Y/PY**, answer questions relating to both baseline and time-varying confounding (1.7 and 1.8) | NA | NA | NA |
| **Questions relating to baseline confounding only** | | | |
| 1.4. Did the authors use an appropriate analysis method that controlled for all the important confounding domains? | PN | PN | PN |
| 1.5. **If Y/PY to 1.4**: Were confounding domains that were controlled for measured validly and reliably by the variables available in this study? | NA | NA | NA |
| 1.6. Did the authors control for any post-intervention variables that could have been affected by the intervention? | PN | PN | PN |
| **Questions relating to baseline and time-varying confounding** | | | |
| 1.7. Did the authors use an appropriate analysis method that controlled for all the important confounding domains and for time-varying confounding? | NA | NA | NA |
| 1.8. **If Y/PY to 1.7**: Were confounding domains that were controlled for measured validly and reliably by the variables available in this study? | NA | NA | NA |
| **Risk of bias judgement** | Critical | Critical | Critical |
| **Bias in selection of participants into the study** | | | |
| 2.1. Was selection of participants into the study (or into the analysis) based on participant characteristics observed after the start of intervention?  **If N/PN to 2.1:** go to 2.4 | N | N | N |
| 2.2. **If Y/PY to 2.1**: Were the post-intervention variables that influenced selection likely to be associated with intervention?  2.3 **If Y/PY to 2.2**: Were the post-intervention variables that influenced selection likely to be influenced by the outcome or a cause of the outcome? | NA  NA | NA  NA | NA  NA |
| 2.4. Do start of follow-up and start of intervention coincide for most participants? | Y | Y | Y |
| 2.5. **If Y/PY to 2.2 and 2.3, or N/PN to 2.4**: Were adjustment techniques used that are likely to correct for the presence of selection biases? | NA | NA | NA |
| **Risk of bias judgement** | Low | Low | Low |
| **Bias in classification of interventions** | | | |
| 3.1 Were intervention groups clearly defined? | Y | Y | Y |
| 3.2 Was the information used to define intervention groups recorded at the start of the intervention? | Y | Y | Y |
| 3.3 Could classification of intervention status have been affected by knowledge of the outcome or risk of the outcome? | N | N | N |
| **Risk of bias judgement** | Low | Low | Low |
| **Bias due to deviations from intended interventions** | | | |
| 4.1. Were there deviations from the intended intervention beyond what would be expected in usual practice? | PN | N | PN |
| 4.2. **If Y/PY to 4.1**: Were these deviations from intended intervention unbalanced between groups *and* likely to have affected the outcome? | NA | NA | NA |
| **Risk of bias judgement** | Low | Low | Low |
| **Bias due to missing data** | | | |
| 5.1 Were outcome data available for all, or nearly all, participants? | PY | Y | Y |
| 5.2 Were participants excluded due to missing data on intervention status? | N | N | N |
| 5.3 Were participants excluded due to missing data on other variables needed for the analysis? | N | N | N |
| 5.4 **If PN/N to 5.1, or Y/PY to 5.2 or 5.3**: Are the proportion of participants and reasons for missing data similar across interventions? | NA | NA | NA |
| 5.5 **If PN/N to 5.1, or Y/PY to 5.2 or 5.3**: Is there evidence that results were robust to the presence of missing data? | NA | NA | NA |
| **Risk of bias judgement** | Low | Low | Low |
| **Bias in measurement of outcomes** | | | |
| 6.1 Could the outcome measure have been influenced by knowledge of the intervention received? | Y | Y | Y |
| 6.2 Were outcome assessors aware of the intervention received by study participants? | Y | Y | Y |
| 6.3 Were the methods of outcome assessment comparable across intervention groups? | Y | Y | PY |
| 6.4 Were any systematic errors in measurement of the outcome related to intervention received? | PN | N | N |
| **Risk of bias judgement** | Moderate | Moderate | Moderate |
| **Bias in selection of the reported result** | | | |
| Is the reported effect estimate likely to be selected, on the basis of the results, from... |  |  |  |
| 7.1. ... multiple outcome *measurements* within the outcome domain? | PN | PN | PN |
| 7.2 ... multiple *analyses* of the intervention-outcome relationship? | PN | PN | PN |
| 7.3 ... different *subgroups*? | PN | PN | PN |
| **Risk of bias judgement** | Moderate | Moderate | Moderate |
| **Overall bias** | | | |
| **Risk of bias judgement** | Critical | Critical | Critical |

1. **Reporting Bias: Funnel Plots by Outcome**

**4. Forest plots for 3-6month follow-up**

*Negative Hedge’s g indicates improvement compared to control.*

1. **Forest plot for Health-Related Quality of Life outcomes at 3-6month follow-up.**

1. **Forest plot for function outcomes at 3-6month follow-up**

**5. Forest plots for subgroup analyses**

12 forest plots are shown: grouped by basis of subgroup analysis (subgroups: intervention type, comparator type, and cognitive diagnosis of participants), for each of HrQoL and function outcomes, at two timepoints: post-intervention, and 3-6month follow-up.

*Negative Hedge’s g indicates improvement compared to control.*

1. **Forest Plot for HrQoL Outcomes Post-Intervention, by Intervention Type**
2. **Forest Plot for Function Outcomes Post-Intervention, by Intervention Type**

1. **Forest Plot for HrQoL Outcomes at Follow-up, by Intervention Type**
2. **Forest Plot for Function Outcomes at Follow-up, by Intervention Type**
3. **Forest Plot for HrQoL Outcomes Post-Intervention, by Comparator Type**
4. **Forest Plot for Function Outcomes Post-Intervention, by Comparator Type**

1. **Forest Plot for HrQoL Outcomes at Follow-up, by Comparator Type**
2. **Forest Plot for Function Outcomes at Follow-up, by Comparator Type**
3. **Forest Plot for HrQoL Outcomes Post-Intervention, by Cognitive Diagnosis of Participants**

1. **Forest Plot for Function Outcomes Post-Intervention, by Cognitive Diagnosis of Participants**

1. **Forest Plot for HrQoL Outcomes at Follow-up, by Cognitive Diagnosis of Participants**
2. **Forest Plot for Function Outcomes at Follow-up, by Cognitive Diagnosis of Participants**
